# A Challenging Case of Fournier’s Gangrene With Multiple Complications

**DOI:** 10.7759/cureus.48036

**Published:** 2023-10-31

**Authors:** Vugar Suleimanov, Kawther Al Hawaj, Fatemah N Al Rebh, Husain Naser, Saud Al Noaim

**Affiliations:** 1 Surgery, Jubail General Hospital, Jubail, SAU; 2 General Surgery, Jubail General Hospital, Jubail, SAU; 3 General Surgery, Jubail General Hospital, Jubail , SAU

**Keywords:** vacuum-assisted wound closure, split thickness skin grafting (stsg), scrotal reconstruction, subphrenic abscess, fournier's gangrene

## Abstract

Fournier's gangrene, a not-so-common urological emergency, is a fast-progressing necrotizing bacterial infection that affects the perineum and external genitalia and can be rapidly fatal unless diagnosed and aggressively managed promptly. Fever, erythematous edema of the scrotum, and palpation of classic scrotal crepitation are among the clinical symptoms. The treatment involves rapid administration of empirical broad-spectrum antibiotics with gram-positive, gram-negative, and anaerobic coverage and rigorous surgical debridement down to the bleeding tissues. The medium-term complications of this condition are primarily associated with extended stay in an intensive care unit and cardiorespiratory, thromboembolic, and cutaneous complications, whereas the long-term complications are mainly functional, aesthetic, and psychological. Also, there are complications inherent to ancillary interventions such as penectomy, orchidectomy, reconstructive surgery, and restoration of digestive continuity. Herein, we present the case of a 40-year-old diabetic male who was admitted with an initial diagnosis of scrotal abscess, which turned out to be Fournier's gangrene. Despite developing multiple complications and numerous surgeries, he made a full recovery and was discharged home after a prolonged hospital stay.

## Introduction

Fournier’s gangrene is a rare, serious condition defined as a type of aggressive necrotizing fasciitis promoted by polymicrobial infection through the genitourinary and perineal regions. It has a high potential to progress rapidly and spread to the lower extremities, abdominal wall, or retroperitoneal space, causing necrosis of tissues and ultimately leading to sepsis, multi-organ failure (MOF), and death. Fournier’s gangrene was discovered in 1764 by Baurienne. Afterward, it was linked to French venereologist Jean Alfred Fournier, who described a series of fatal cases of idiopathic progressive gangrene of the genitalia with a sudden onset in five young men in 1883 [1].

Currently, it has been found to happen at any age, even in children, and in any gender affected by various recognizable factors [2]. Male sex remains a risk factor for Fournier’s gangrene, with a male/female ratio of 10:1 [3]. The mortality rate is high (20% to 40%), with some authors reporting it to be up to 80% [4]. The most common risk factors for Fournier’s gangrene are diabetes mellitus, alcoholism, malnutrition, smoking, malignancies, immunosuppressive therapy, and obesity. Invasive anorectal or urogenital procedures, abscesses, penile intravenous drug use, trauma, and burns are well-known causes that can lead to Fournier's gangrene in susceptible individuals.

Delayed management of Fournier’s gangrene is known to lead to sepsis, MOF, and disseminated intravascular coagulation (DIC). The initial treatment includes urgent resuscitation, broad-spectrum antibiotics, and immediate surgical debridement to reduce systemic toxicity and stop the rapid spread of the infectious progress [5]. Our report describes the case of a diabetic male patient with Fournier’s gangrene, complicated by extension to the abdominal wall, pelvis, lower back, pleural effusion, and subdiaphragmatic abscess.

## Case presentation

A 40-year-old diabetic male presented to the emergency department of a district general hospital with fever, scrotal pain, and swelling of 10 days duration. He was admitted to the surgical ward as a case of scrotal abscess, and debridement of necrotic tissues was performed in the operating room (OR) by a urologist. Broad-spectrum antibiotics were started, and blood sugar control, fluid-electrolyte balance, and acid-base balance were addressed. The general surgery team was consulted on the second day of admission for abdominal wall erythema and induration on the right side.

**Figure 1:**
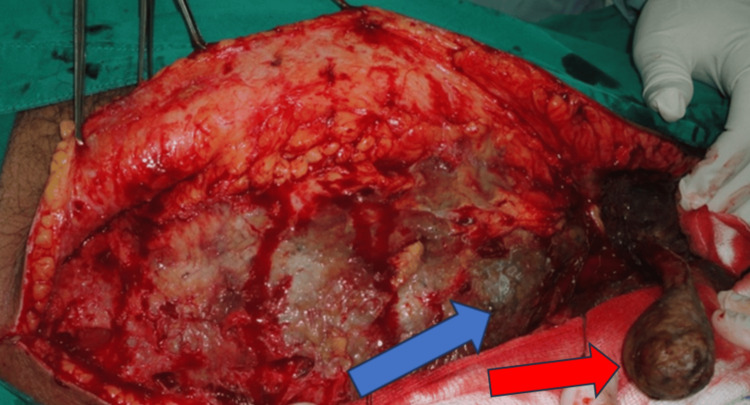
Initial incision through the skin and subcutaneous tissues revealed necrotic fascia over the abdominal wall (blue arrow). Covering of the right testis is necrotic too (red arrow).

On examination, we realized that the infection had spread to the abdominal wall through the right groin, up to the anterior chest wall on the right side. Skin crepitus was present along with induration and erythema over a large area of the abdominal wall on the right side. Needle aspiration of the involved area revealed dishwater-colored fluid. The patient was diagnosed with necrotizing fasciitis (NF), and he was taken back to the OR. Upon making the skin incision, necrotic fascia emerged (Figure 1), and extensive debridement of involved areas (right side of the abdominal wall, retroperitoneal tissues, lower back) was carried out. A swab was taken for culture and sensitivity testing.

**Figure 2:**
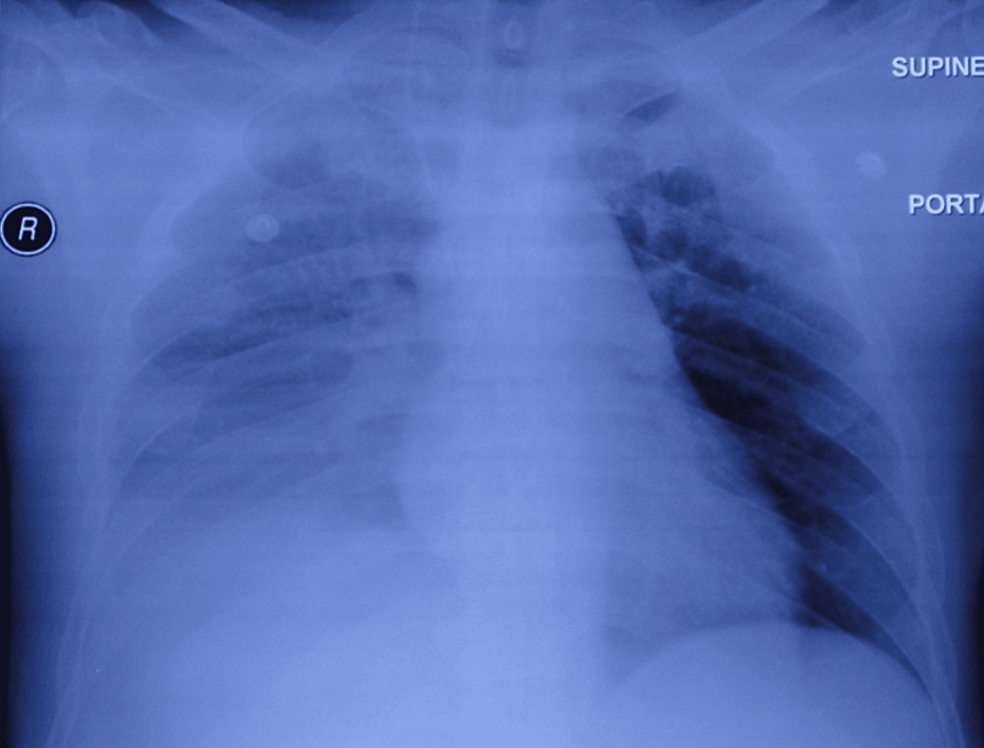
Right pleural effusion

Antibiotics were changed to piperacillin/tazobactam 4.5 gm every six hours and clindamycin injection 900 mg every eight hours intravenously (on admission, he was taking ceftriaxone and metronidazole). The patient was shifted to the ICU, and a repeat debridement was carried out the next day. The patient continued to have a high fever, and his white blood cell count (WBC) remained high. It took several more trips to the OR to have complete control of the infection. The wound swab grew multiple multidrug-resistant organisms, such as group A beta-hemolytic *Streptococcus* (GABHS), *Escherichia coli*, and *Enterococci*. Antibiotics were changed according to sensitivity patterns, and vacuum-assisted closure (VAC) for the wound was employed as well. The patient gradually showed signs of improvement; his fever and WBC normalized. Ten days into admission, the patient started to deteriorate again, with a high fever (over 40 °C) and the new onset of leukocytosis. The chest X-ray showed a large right-sided pleural effusion (Figure 2), but drainage of the effusion did not help to bring down the relentless fever.

**Figure 3:**
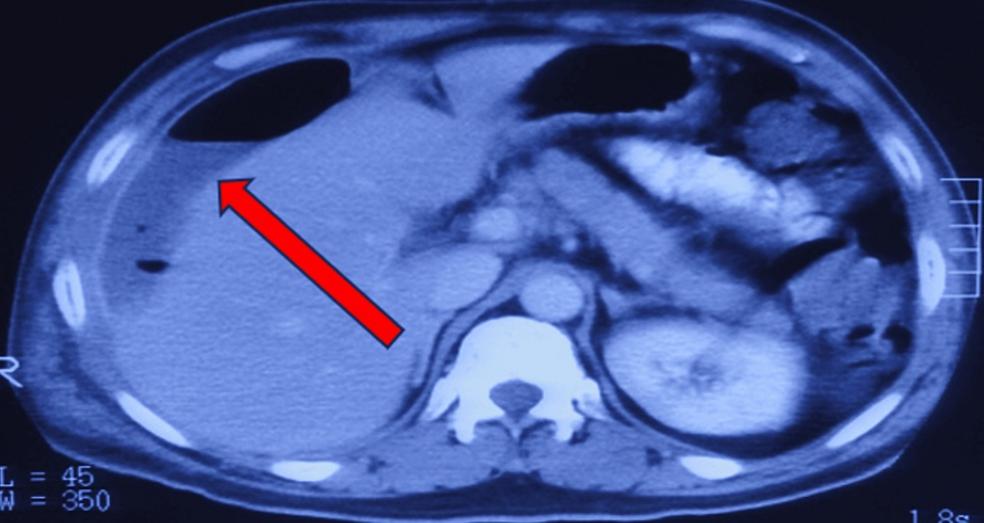
Right subphrenic abscess (red arrow) on CT

A septic workup did not help find the source of the infection. At this time, the well-known aphorism of "pus somewhere, pus nowhere else, and pus under the diaphragm" came into play. A contrast-enhanced single-energy computed tomography (SECT) of the chest, abdomen, and pelvis revealed a large right subphrenic abscess (Figure 3).

**Figure 4:**
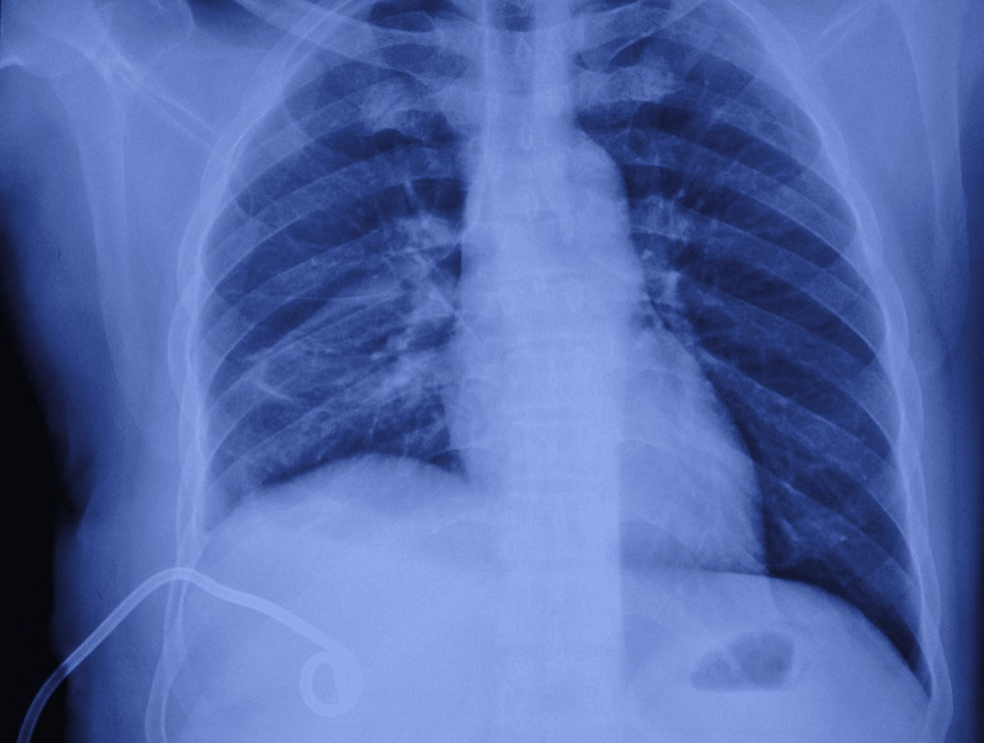
Pigtail drain in subdiaphragmatic space (bottom left corner)

Ultrasound-guided drainage of the subphrenic abscess was carried out by a 14Fr pigtail catheter (Figure 4) on day 14 of admission. The patient improved gradually after the drainage of the subphrenic abscess; the pus culture grew *E. coli*, *Pseudomonas aeruginosa*, and *Acinetobacter baumannii*. Again, antibiotics were changed based on sensitivity patterns. Gradually, the fever settled, the wounds started to granulate and repeat cultures came back negative.

**Figure 5:**
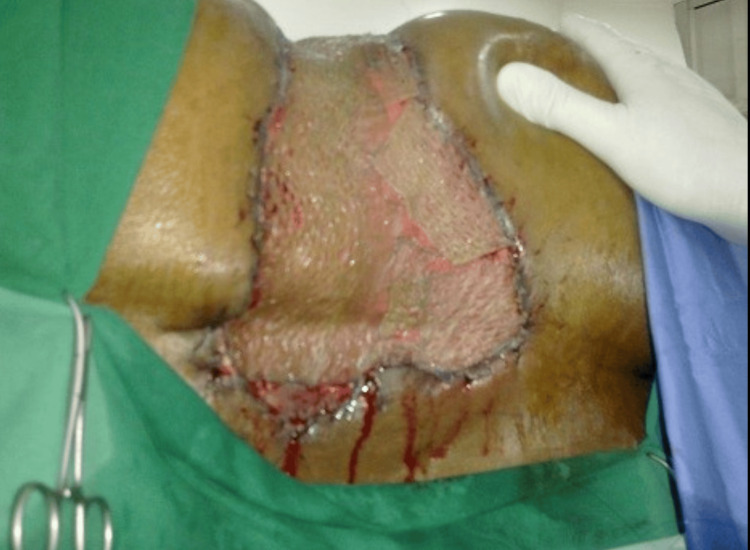
Intraoperative view of the split-thickness skin grafting

The drain was removed after 12 days, and the decision was made to carry out split-thickness skin grafting. The patient was taken to the OR again on day 34 of admission, and a split-thickness skin graft was harvested from the anterior surfaces of both thighs by using an Aesculap electric dermatome (Aesculap Inc., Center Valley, PA, USA). The harvested skin was meshed to increase the surface area and applied to the prepared recipient area, fixed by continuous polypropylene sutures (Figure 5).

**Figure 6:**
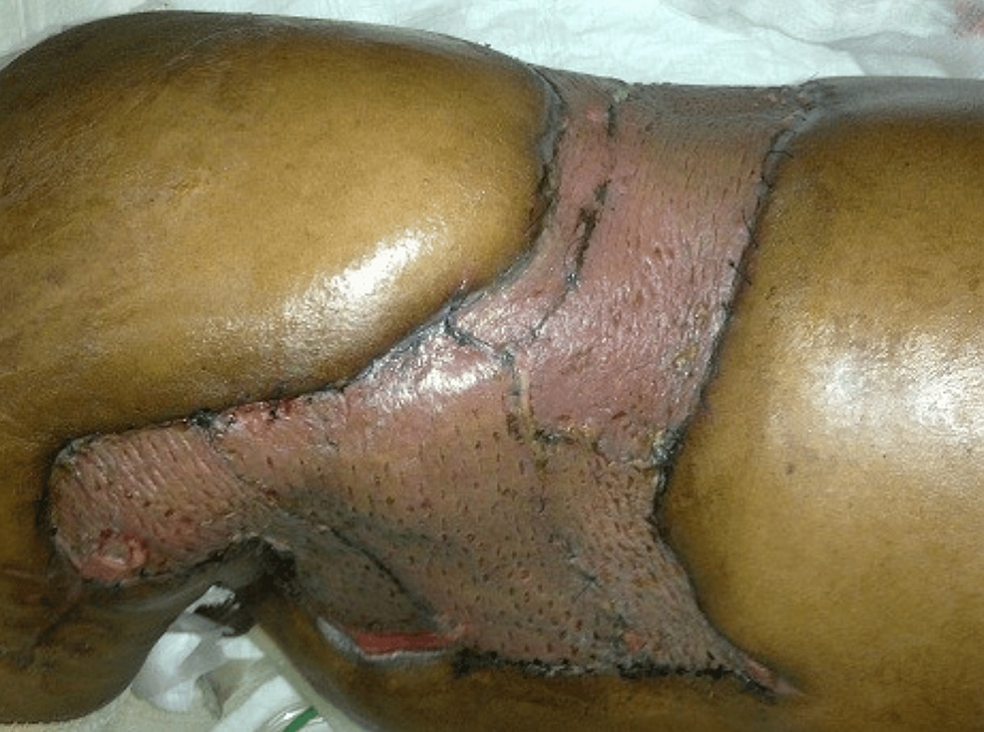
Postoperative view of the skin graft showing viability in all grafted areas

The patient had an uneventful recovery after the surgery, and on day 5, the dressing was changed. The graft was viable in all grafted areas (Figure 6). The donor sites were healing well too. Sutures were removed later on, and the patient was counseled regarding various possibilities of scrotal reconstruction since he had "shamefully exposed" testicles, like in most cases of Fournier's gangrene.

**Figure 7:**
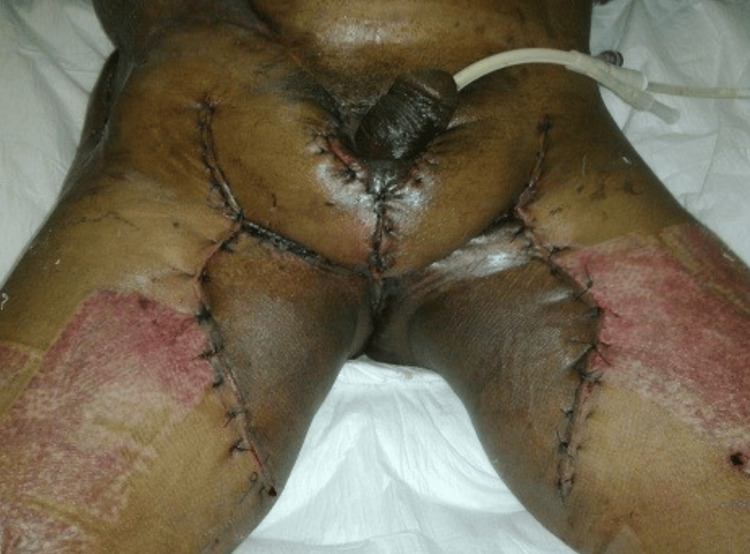
Successful scrotal reconstruction using anteromedial thigh fasciocutaneous flaps

The decision was made to perform a scrotal reconstruction using anteromedial thigh fasciocutaneous flaps on both sides after discussing various options with the patient. The procedure was carried out successfully on day 46 of admission. There was no flap necrosis or infection after the surgery, which are the two most common complications after such a procedure (Figure 7). The patient was discharged home in good condition a few days after scrotal reconstruction. The total hospital stay was 52 days.

The patient was followed up in the outpatient clinic one week, one month, and six months, respectively, after discharge. He remained well except for the development of an incisional hernia, which was anticipated. The incisional hernia was repaired too by using a polypropylene mesh, which was successful.

## Discussion

Fournier's gangrene is a polymicrobial infection involving aerobic and anaerobic bacteria. The most common organisms isolated include *E. coli*, *Streptococcus* species, and anaerobic bacteria such as *Bacteroides fragili*s. The condition arises from a synergistic infection, often originating from a genitourinary source or skin injury. Comorbidities such as diabetes and immunosuppression contribute to the development and severity of the disease. Prompt recognition of clinical signs, along with early aggressive surgical debridement, is crucial for optimal outcomes [6].

In this case, the initial diagnosis of a scrotal abscess, coupled with the patient's underlying diabetes and lack of treatment, placed him at higher risk for Fournier's gangrene. Despite urgent intervention, the infection rapidly spread, necessitating an extensive and aggressive debridement procedure. The involvement of multiple anatomical regions highlights the aggressive and life-threatening nature of Fournier's gangrene. The shift in antibiotic therapy to cover a broader spectrum of pathogens, including both aerobic and anaerobic bacteria, ensures adequate treatment coverage [7]. The rapid progression of this patient's scrotal abscess to involve the perineum, abdominal wall, and pelvic region highlights the aggressive nature of infections in diabetic individuals.

Diabetic patients are predisposed to infections due to immunosuppression and impaired defense mechanisms against invading organisms, making them particularly susceptible to severe and polymicrobial infections. In this case, cultures revealed GABHS, *E. coli*, and *Enterococci *as the initial pathogens. This demonstrates the importance of obtaining cultures and tailoring antibiotic therapy accordingly to ensure effective treatment [8]. Including high-dose clindamycin in antibiotic therapy has been shown to be beneficial in necrotizing soft tissue infections (NSTI) by multiple studies, especially if GABHS is one of the causative organisms. The beneficial effects of clindamycin have been attributed to reducing the synthesis of penicillin-binding proteins and bacterial toxins, suppressing lipopolysaccharide-induced mononuclear synthesis of tumor necrosis factor-α (TNF-α), and others [9].

The utilization of VAC therapy in the management of the patient's wound underscores its effectiveness in promoting wound healing and reducing the risk of complications [10]. Our patient showed improvement after the initiation of VAC therapy, but despite initial improvement, he later developed a high fever and leukocytosis de novo, suggesting the presence of infection somewhere else. Although rare, especially in cases of Fournier’s gangrene, the search for infection should include looking for a subphrenic abscess, which proved to be the case in our patient [10]. The subsequent identification of *E. coli, P. aeruginosa*, and* A. baumannii* in the subphrenic abscess highlights the need for appropriate sensitivity-guided antibiotic therapy. Continuous monitoring and adjustment of antibiotic therapy is crucial in managing complex infections to ensure optimal outcomes [11].

In almost all cases of Fournier’s gangrene, if the patient recovers from a life-threatening infection, the treating surgeon must deal with the "shamefully" exposed testes. There are multiple options to cover exposed testes: split-thickness skin grafting, utilization of various flaps, placing the testes in subcutaneous pouches on ipsilateral thighs, etc. [12-15]. The pros and cons of each option must be discussed with the patient, and the most suitable method must be utilized, considering patient factors, preferences, availability of expertise, etc. We chose an anteromedial thigh fasciocutaneous flap reconstruction after discussing it with the patient, which was carried out successfully.

## Conclusions

Fournier’s gangrene has a high mortality rate in immunocompromised patients. Maintaining a high index of suspicion for Fournier’s gangrene in patients presenting with scrotal or perineal infection and early, aggressive, and repeated surgical debridement, along with the administration of a high dose of broad-spectrum antibiotics, can improve patient outcomes. Including clindamycin in the antibiotic regimen, especially if GABHS is grown from cultures, is recommended to reduce the spread of infection.

Vacuum-assisted closure therapy offers significant benefits in complex wound management, aiding in the removal of infected tissue, promoting healing, reducing the risk of complications, and reducing the length of hospital stay. Although rare and not reported in Fournier’s gangrene cases before, if the patient has a relentless fever and septic work-up is not helpful to find the source, we recommend considering a subdiaphragmatic abscess in the differential diagnosis and a CT of the chest, abdomen, and pelvis. Scrotal reconstruction can be carried out using a variety of techniques, but one should choose an approach that is tailored to the patient’s needs and preferences, coupled with available expertise.
